# Supplementary material for: QCR7 affects the virulence of Candida albicans and the uptake of multiple carbon sources present in different host niches
Source: Front Cell Infect Microbiol. 2023 Feb 27;13:1136698. doi: 10.3389/fcimb.2023.1136698 (PMC10009220; doi:10.3389/fcimb.2023.1136698)
Supplement: Supplementary file 13 [file Table_1.pdf]

Table S1.Strains used in this study

Strain	Relevant Genotype	Full Genotype	Reference
SN152		arg4Δ/arg4Δ, leu2Δ/leu2Δ, hisΔ/hisΔ ,URA3/ura3Δ::imm434IRO1/iro1Δ::imm434	Noble et al., 2005
SN250	Wild type	*leu2Δ::C.m.LEU2/leu2Δ::C.d.HIS1, his1Δ/his1Δ, arg4Δ/arg4Δ, leu2D/leu2Δ ura3Δ/URA3, iro1Δ/IRO1*	Noble et al., 2010
NC301	*qcr7Δ/Δ*	*qcr7Δ::C.m.LEU2/qcr7Δ::C.d.HIS1, his1Δ/his1Δ, arg4Δ/arg4Δ, leu2Δ/leu2Δ, ura3Δ/URA3, iro1Δ/IRO1*	this study
NC302	*QCR7-complemented*	*leu2Δ::QCR7::C.d.ARG4/leu2Δ::C.d.HIS1, his1Δ/his1Δ, arg4Δ/arg4Δ, leu2Δ/leu2Δ, ura3Δ/URA3, iro1Δ/IRO1*	this study
NC303	*HWP1*^{OE},*qcr7Δ/Δ*	*qcr7Δ::C.m.LEU2/qcr7Δ::C.d.HIS1, his1Δ/his1Δ, arg4Δ/arg4Δ, leu2Δ/leu2Δ, ura3Δ/URA3, iro1Δ /IRO1,NEUT5L/NEUT5L::pADH-HWP1-ARG4*	this study
NC304	*YWP1*^{OE},*qcr7Δ/Δ*	*qcr7Δ::C.m.LEU2/qcr7Δ::C.d.HIS1, his1Δ/his1Δ, arg4Δ/arg4Δ, leu2Δ/leu2Δ, ura3Δ/URA3, iro1Δ /IRO1,NEUT5L/NEUT5L::pADH-YWP1-ARG4*	this study
NC305	*XOG1*^{OE},*qcr7Δ/Δ*	*qcr7Δ::C.m.LEU2/qcr7Δ::C.d.HIS1, his1Δ/his1Δ, arg4Δ/arg4Δ, leu2Δ/leu2Δ, ura3Δ/URA3, iro1Δ /IRO1,NEUT5L/NEUT5L::pADH-XOG1-ARG4*	this study
NC306	*SAP6*^{OE},*qcr7Δ/Δ*	*qcr7Δ::C.m.LEU2/qcr7Δ::C.d.HIS1, his1Δ/his1Δ, arg4Δ/arg4Δ, leu2Δ/leu2Δ, ura3Δ/URA3, iro1Δ /IRO1,NEUT5L/NEUT5L::pADH-SAP6-ARG4*	this study
NC307	*HYR1*^{OE},*qcr7Δ/Δ*	*qcr7Δ::C.m.LEU2/qcr7Δ::C.d.HIS1, his1Δ/his1Δ, arg4Δ/arg4Δ, leu2Δ/leu2Δ, ura3Δ/URA3, iro1Δ /IRO1,NEUT5L/NEUT5L::pADH-HYR1-ARG4*	this study
NC308	QCR7^{OE},*bcr1Δ/Δ*	*BCR1Δ::C.m.LEU2/BCR1Δ::C.d.HIS1, his1Δ/his1Δ, arg4Δ/arg4Δ, leu2Δ/leu2Δ, ura3Δ/URA3, iro1Δ /IRO1,NEUT5L/NEUT5L::pADH-QCR7-ARG4*	this study
NC309	QCR7^{OE},*brg1Δ/Δ*	*BRG1Δ::C.m.LEU2/BRG1Δ::C.d.HIS1, his1Δ/his1Δ, arg4Δ/arg4Δ, leu2Δ/leu2Δ, ura3Δ/URA3, iro1Δ /IRO1,NEUT5L/NEUT5L::pADH-QCR7-ARG4*	this study
NC310	QCR7^{OE},*ndt80Δ/Δ*	*NDT80Δ::C.m.LEU2/NDT80Δ::C.d.HIS1, his1Δ/his1Δ, arg4Δ/arg4Δ, leu2Δ/leu2Δ, ura3Δ/URA3, iro1Δ /IRO1,NEUT5L/NEUT5L::pADH-QCR7-ARG4*	this study
NC311	QCR7^{OE},*rob1Δ/Δ*	*ROB1Δ::C.m.LEU2/ROB1Δ::C.d.HIS1, his1Δ/his1Δ, arg4Δ/arg4Δ, leu2Δ/leu2Δ, ura3Δ/URA3, iro1Δ /IRO1,NEUT5L/NEUT5L::pADH-QCR7-ARG4*	this study
NC312	QCR7^{OE},*efg1Δ/Δ*	*EFG1Δ::C.m.LEU2/EFG1Δ::C.d.HIS1, his1Δ/his1Δ, arg4Δ/arg4Δ, leu2Δ/leu2Δ, ura3Δ/URA3, iro1Δ /IRO1,NEUT5L/NEUT5L::pADH-QCR7-ARG4*	this study
NC313	QCR7^{OE},*tec1Δ/Δ*	*TEC1Δ::C.m.LEU2/TEC1Δ::C.d.HIS1, his1Δ/his1Δ, arg4Δ/arg4Δ, leu2Δ/leu2Δ, ura3Δ/URA3, iro1Δ /IRO1,NEUT5L/NEUT5L::pADH-QCR7-ARG4*	this study
