# Supplementary material for: QCR7 affects the virulence of Candida albicans and the uptake of multiple carbon sources present in different host niches
Source: Front Cell Infect Microbiol. 2023 Feb 27;13:1136698. doi: 10.3389/fcimb.2023.1136698 (PMC10009220; doi:10.3389/fcimb.2023.1136698)
Supplement: Supplementary file 14 [file Table_2.pdf]

Table S2.Primes used in this study		
primer name	*purpose*	*sequence*
Qcr7 primer 1	primer for *QCR7* 5'flank,forward	*CCATATGCTGAAACTCTTTTCC*
Qcr7 primer 3	primer for *QCR7* 5'flank,reverse	*cacggcgcgcctagcagcgg*
Universal primer 2	common primer for *Leu2/His1/Arg4* 5'flank,forward	*gcagggatgcggccgctgac*
Universal primer 5	common primer for *Leu2/His1/Arg4* 5'flank,reverse	*ccgctgctaggcgcgccgtg AGCTCGGATCCACTAGTAACG*
Qcr7 primer 4	primer for *QCR7* 3'flank,forward	*gtcagcggcgcatccctgcGTAAGTAAGAGGAGGAG*
Qcr7 primer 6	primer for *QCR7* 3'flank,reverse	*GGGACCTCATACAATTGTAACG*
Qcr7 upstream check	primer for *QCR7* 5'flank check, forward	*GGACCAGAAAGTGGAGCATCTC*
Universal Leu left	*Leu2* ORF check, reverse	*GGGTTTGGGTAGTGAATAAAGG*
Universal Leu right	*Leu2* ORF check, forward	*CATCATTTAATCGGTGGTGC*
Qcr7 downstream check	primer for *QCR7* 3'flank check, reverse	*GCTGGATTAGTGGCTACTACAG*
Universal His left	*His1* ORF check, reverse	*CTTGAGCATATCCCATGGTCTAG*
Universal His right	*His1* ORF check, forward	*CGGCTCAGGAATATGTCTTGTG*
Qcr7 left	*QCR7* ORF check, reverse	*CTATGACATCTGTCGTTAAGGC*
Qcr7 right	*QCR7* ORF check, forward	*ATAGCAGTTTGCATAATTGGGG*
Qcr7 primer13	primer for *QCR7* ORF to reconstitute, reverse	*cacggcgcgcctagcagcggTTACGCTTTAACTTCAATATTA*
Universal Arg AB right	*ARG4* ORF check, reverse	*CTTCCATTGTGAAGATCGTCTG*
Universal Arg AB left	*ARG4* ORF check, forward	*CAATCCTGGGTTAGAAACACCC*
XTO1030(18S rRNA-F)	qPCR for *18S*, forward	*CGCAAGGCTGAAACTTAAAGG*
XTO1031(18S rRNA-R)	qPCR for *18S*, reverse	*AGCAGACAAATCACTCCACC*
XTO1084(Als1-F)	qPCR for *ALS1*, forward	*TGCTACAACCACCACTGTTAC*
XTO1085(Als1-R)	qPCR for *ALS1*, reverse	*GGCATAGGATTGTGACCAGTATTC*
XTO1088 (HWP1-F)	qPCR for *HWP1*, forward	*ACCACTTCAGAATCATCATC*
XTO1089 (HWP1-R)	qPCR for *HWP1*, reverse	*AACACCAGTAGTAACTTCAC*
XTO1090(XOG1-F)	qPCR for *XOG1*, forward	*ACGAATATCACTGGACACAA*
XTO1091(XOG1-R)	qPCR for *XOG1*, reverse	*ATACTGAACTTGACCTTGGA*
XTO1092(HYR1-F)	qPCR for *HYR1*, forward	*ACATCAAGTCCTGGTCAATC*
XTO1093(HYR1-R)	qPCR for *HYR1*, reverse	*ATGGAACAGTGGTGAAGATAG*
XTO1094(YWP1-F)	qPCR for *YWP1*, forward	*AGTCATCACCATCACTTCAT*
XTO1095(YWP1-R)	qPCR for *YWP1*, reverse	*TCAGTTACAACACCTTCAGA*
XTO1096(PGA7-F)	qPCR for *PGA7*, forward	*GGCTCAATCAACAACTACAG*
XTO1097(PGA7-R)	qPCR for *PGA7*, reverse	*GCAGAAGATGAAGGAGATGA*
XTO1102(Als3-F)	qPCR for *ALS3*, forward	*AATAATGGTGGTGGTAATGC*
XTO1103(Als3-R)	qPCR for *ALS3*, reverse	*TTGAGATTGGTTGGTTGATG*
XTO1104 (CSH1-F)	qPCR for *CSH1*, forward	*CGATACTGCTGATACTTACTC*
XTO1105 (CSH1-R)	qPCR for *CSH1*, reverse	*CTCAATCCTTTACCGTTCAT*
